# The 3,000 rice genomes project: new opportunities and challenges for future rice research

**DOI:** 10.1186/2047-217X-3-8

**Published:** 2014-05-28

**Authors:** Jia-Yang Li, Jun Wang, Robert S Zeigler

**Affiliations:** 1 Chinese Academy of Agricultural Sciences, 12 S. Zhong-Guan-Cun St, Beijing 100081, China; 2 BGI, Bei Shan Industrial Zone, Yantian District, Shenzhen 518083, China; 3 International Rice Research Institute, DAPO 7777, Metro Manila 1301, Philippines

**Keywords:** *Oryza sativa*, Genetic resources, Genome diversity, Phenomics, Sequencing

## Abstract

Rice is the world’s most important staple grown by millions of small-holder farmers. Sustaining rice production relies on the intelligent use of rice diversity. The 3,000 Rice Genomes Project is a giga-dataset of publically available genome sequences (averaging 14× depth of coverage) derived from 3,000 accessions of rice with global representation of genetic and functional diversity. The seed of these accessions is available from the International Rice Genebank Collection. Together, they are an unprecedented resource for advancing rice science and breeding technology. Our immediate challenge now is to comprehensively and systematically mine this dataset to link genotypic variation to functional variation with the ultimate goal of creating new and sustainable rice varieties that can support a future world population that will approach 9.6 billion by 2050.

## Background

Rice (*Oryza sativa* L.) is the staple food for half the world population, particularly for the poorest of Asia. Rice played a major role in the Green Revolution in the 1960s, a well-known story of modern plant breeding that contributed significantly to global food security. Now, rice production must increase by at least 25% by 2030 to keep pace with predicted population growth. This has to be achieved using less land, less water and under more severe environmental stresses expected, due to the effects of climate change and disease pressures. Much of this increase must come from genetic improvement of rice. Rice research has progressed greatly in the past years, highlighted by the completion of the first high-quality rice genome in 2005 [1], that stimulated global efforts in rice functional genomics research [2,3]. However, the information from rice genetics and genomics research has yet to fundamentally change rice breeding practices. The currently available sequence data are not yet in a form readily usable by most rice breeders nor is the global community yet prepared to manage data influxes that may well be orders of magnitude greater than previously encountered.

## Exploring rice genetic diversity

Rice is known for tremendous within-species and within-genus genetic diversity, both critical foundations for rice improvement. This rich source of genetic diversity is preserved in more than 230,000 germplasm accessions of *Oryza* maintained in genebanks worldwide, mostly of Asian origin. Exploring this diversity at the sequence level has been, until recently, only a dream of rice scientists. But two years ago, the Chinese Academy of Agricultural Sciences (CAAS), the Beijing Genomics Institute (BGI) Shenzhen and the International Rice Research Institute (IRRI), launched a program to systematically sequence a broad spectrum of known diversity across the species. “The 3,000 (3K) Rice Genomes Project” is a major step towards revealing the genomic diversity in all of the world’s rice germplasm collections. But for this ambitious effort to be meaningful beyond the scientific community, significant investments will have to be made in measuring plant performance under a wide range of conditions, as well as the development of data management approaches that can apply the genetic knowledge to practical uses by extracting genotype-to-phenotype relationships for a better understanding of plant biology.

## Current status and plans

The 3K Rice Genomes Project has completed sequencing 3,000 rice genomes with an average sequencing depth of 14×. The panel of sequenced rice accessions represents a diverse set originating from 89 countries, and were selected from a combined collection of ~13,000 *O. sativa* accessions from the ~180,000 rice accessions conserved in the International Rice Genebank Collection (IRGC) at IRRI [4] and the China National Crop Genebank (CNCG) [5]. The 3K lines included most rice mega-varieties being grown across large areas of different ecosystems throughout Asia [6]. The parental lines of popular varieties and selected genetic mapping populations were also included; 400 of which were parental lines for genome-wide introgression lines for multiple complex traits developed using novel molecular breeding strategies [7]. While this approach should capture most of the genetic variation in rice, a further round of sampling based on insights obtained from the 3K project will be needed to capture unusual and possibly highly useful variants [8].

The sequence data for the 3K rice genomes, deposited in the *GigaScience* journal database, *GigaDB*[9], provides an unprecedented resource for rice scientists. Not only will this giga-dataset form the basis for advancing our understanding of rice’s history of selection (natural or imposed), it provides the platform for large-scale discovery of genetic variation associated with important traits for breeding applications [7]. While scientists are thrilled with the potential impact of the project, many challenges remain to integrate the sequence data with genomic, genetic and phenotypic data from many sources. A rice diversity information portal and underlying databases will be needed to facilitate large-scale gene/trait discovery and allele mining [10], enable the development of new molecular breeding strategies [7], and inform a strategy for better conserving rice genetic resources [11]. There are several discrete steps that are necessary in order for the outcomes of the 3K Rice Genomes Project to have practical applications:

1) Decipher global and local population differentiation;

2) Construct new high-quality reference genomes representing major varietal groups;

3) Create haplotype maps by linkage disequilibrium and recombination break-point analyses;

4) Build one or more pan-genome assemblies for each of the varietal groups and create annotation mappings between pan-genomes;

5) Discover single nucleotide polymorphisms (SNPs), structural variants and indels between and within populations.

While the applications are clear and the opportunities nearly boundless, the analytical challenges are indeed enormous. Sequence information, while offering bountiful material for evolutionary studies, offers little on its own for practical applications for rice breeders. Plans are being developed with multiple institutions under the auspices of the Global Rice Science Partnership (GRiSP) of the Consultative Group on International Agricultural Research for extensive and systematic characterization of phenotypes of accessions for a wide range of traits to discover important sequences and regions using genome-wide association studies. Phenotyping, coordinated by IRRI and CAAS, is in progress for biotic and abiotic stress tolerance, grain quality characters, plant development and yield traits. High-throughput phenomics using image and sensor capture from controlled environment and field-based (ambient and managed) platforms will contribute immensely to the ability to associate sequence information with phenotypes. This combined effort will provide greater depth and coverage compared to prior studies yielding deeper insights and broader applications.

Even before phenotypic data becomes available, we expect that analyses of the 3K rice genomes data will yield useful information, and that greater sequencing depth or higher sampling will be guided by analysis of the population structure. And, as multiple high-quality reference genomes are developed, many more SNPs in the pan-rice genome should be discovered. The emerging high-density maps will further facilitate efficient gene discovery and allele mining. Thus, an early outcome of the 3K Rice Genomes Project will be new population-specific genotyping arrays useful to a wide range of genetic and breeding applications. Secondly, detailed studies should reveal population structures that have been shaped by evolutionary, domestication and selection processes. Identification and detailed analyses of unique cryptic structural genomic variants across the rice genome will allow us to understand their contributions to the previously identified varietal groupings in rice. Thirdly, by including lines used in mapping and breeding programs, we can target gene validation for direct use for trait improvement in breeding populations. Breeding populations developed from the sequenced lines will enable implementation, testing and improvement of novel breeding strategies, such as genomic selection and recurrent selection in rice breeding programs [6].

## Challenges

Completion of the sequencing and preliminary analyses of 3K rice genomes is just the first step in establishing an information platform of integrated databases and advanced tools to accelerate rice breeding. This effort will be similar in scope to the development of the Arabidopsis Information Portal (AIP) [12]. IRRI has initiated the International Rice Informatics Consortium (IRIC) under GRiSP. While writing this paper, discussions are underway to formalize the consortium agreement for IRIC and technical aspects of the portal design, standards for meta-data for interoperability, and persistent, diagnostic germplasm identifiers. First targets include curation of 3K rice genomes data and other public data, definition of reference genomes, design and archival of phenotyping datasets, and a web-based interface, or portal, and tools for population structure, genome-wide association studies and diversity browsing. Still, linking diversity in the 3K rice genomes dataset to phenotypic variation and environmental adaptation requires a long-term global effort in rice functional genomics research. For a more complete understanding of *O. sativa* genetic diversity and genes underlying important rice traits, future research should not only focus on identifying and characterizing rare genes/alleles with large effect, but also on novel allelic combinations underpinning complex traits. With such value-added information integrated into the database and access to appropriate tools through the Web portal, a more systematic discovery and enhanced utilization of rich genetic diversity will be possible [7,10,11]. While this project will undoubtedly stimulate another round of rapid advances in rice genetics, numerous challenges exist to extract the most information from the sequence and phenomics data to establish a global, public information platform useful not only for experimental research, but also for practical rice breeding. These challenges will be overcome through global rice research efforts to ensure scientific advancements and delivery of benefits for rice farmers and to maintain the food security of humankind. The challenge is large and will require unprecedented collaboration that transcends national, institutional and personal ambitions.

## Endnote

^a^“Mega-varieties” refers to those varieties that have been grown on at least 1 M hectares.

## Abbreviations

3K: The 3000 Rice Genomes Project; CAAS: Chinese Academy of Agricultural Sciences; CNCG: China National Crop Genebank; GRiSP: Global Rice Science Partnership; IRIC: International Rice Informatics Consortium; IRRI: International Rice Research Institute; IRGC: International Rice Genebank Collection; SNP: Single Nucleotide Polymorphism.

## Competing interests

The authors declare that they have no competing interests.

## Authors’ contributions

JYL, JW and RSZ wrote the article. All authors have read and approved the final manuscript.
